# Cryo-EM structure of the interleukin-31 receptor complex defines non-canonical interactions that confer receptor specificity

**DOI:** 10.1016/j.jbc.2026.113472

**Published:** 2026-08-24

**Authors:** Yong Feng, Gaofei Qian, Zichu Wei, Xianchi Dong

**Affiliations:** 1 Department of Oncology, Nanjing Drum Tower Hospital, State Key Laboratory of Pharmaceutical Biotechnology, School of Life Sciences, Nanjing University, Engineering Research Center of Protein and Peptide Medicine, Ministry of Education, Nanjing, China; 2 Institute of Artificial Intelligence Biomedicine, Nanjing University, Nanjing, China

**Keywords:** interleukin-31, receptor complex, cryo-electron microscopy, receptor-specific recognition

## Abstract

Interleukin-31 (IL-31) is a Th2-associated cytokine that induces inflammatory and pruritic diseases through a heterodimeric receptor composed of IL-31Rα and OSMRβ. Although IL-31 has emerged as an important therapeutic target, the molecular mechanism by which IL-31 engages IL-31Rα and OSMRβ to assemble its receptor complex remains poorly defined. Here we report a 3.3 Å cryo-EM structure of the human IL-31/IL-31Rα/OSMRβ complex. The structure reveals that IL-31 assembles its receptors through a site 2–site 3 architecture reminiscent of LIF and OSM, yet employs distinct receptor-recognition features at both interfaces. Site 2 is dominated by a hydrophilic IL-31–IL-31Rα interface that lacks the aromatic anchoring mode observed in LIF and OSM receptor complexes, whereas site 3 adopts a remodeled anchoring mode involving IL-31 K134, T130, and the conserved OSMRβ W267 anchor. Structure-guided mutagenesis and SPR binding analyses support the contributions of site 2 and site 3 interface residues to receptor recognition. Together, these findings reveal how non-canonical interactions at sites 2 and 3 confer receptor specificity within the IL-31 receptor complex.

Interleukin-31 (IL-31) is a pleiotropic cytokine predominantly produced by activated Th2 CD4^+^ T cells and plays a central role in the regulation of neuroimmune and inflammatory responses (1, 2). IL-31 has been implicated in the pathogenesis of multiple diseases, particularly atopic dermatitis (1, 3), chronic pruritus (4), and cancer-related immune regulation (5, 6), making the IL-31 signaling axis an attractive target for immunotherapy. IL-31 activates downstream signaling pathways by engaging two cell-surface receptors, IL-31Rα and OSMRβ (1, 7). These receptors are expressed in a broad range of immune cells (including T cells, eosinophils, and macrophages) as well as non-immune cells (such as keratinocytes, epithelial cells, and dorsal root ganglion neurons) (8, 9, 10). Binding of IL-31 to its receptor complex triggers multiple intracellular pathways, including mitogen-activated protein kinase (MAPK), phosphoinositide 3-kinase/protein kinase B (PI3K/AKT), and Janus kinase/signal transducer and activator of transcription (JAK/STAT) signaling (2, 7). Accordingly, several therapeutic agents targeting IL-31 or its receptors have been developed, among which nemolizumab, a humanized monoclonal antibody against IL-31Rα, has been shown to improve pruritus in patients with atopic dermatitis (11, 12, 13).

IL-31 is a member of the IL-6 family of cytokines, which also includes IL-6, IL-11, ciliary neurotrophic factor (CNTF), cardiotrophin-like cytokine factor 1 (CLCF1), leukemia inhibitory factor (LIF), oncostatin M (OSM), and cardiotrophin-1 (CT-1) (14, 15). For many IL-6 family cytokines, receptor assembly involves the shared signal-transducing receptor glycoprotein 130 (gp130), which serves as a central scaffold for receptor assembly and downstream signaling (15). Structural studies of gp130-containing receptor complexes have provided important insights into the molecular mechanisms governing cytokine recognition and receptor activation within the IL-6 family (16, 17, 18). While receptor assembly by IL-6 and IL-11 involves α-receptor engagement at site 1 (17, 19), structurally characterized LIF and OSM receptor complexes assemble without a classical site 1 α-receptor module and instead rely primarily on site 2 and site 3 receptor recruitment (16, 18). In contrast to other characterized IL-6 family cytokines, IL-31 does not utilize the shared signaling receptor gp130 and instead signals through a heterodimeric receptor complex composed of IL-31Rα and OSMRβ. The structural basis underlying how IL-31Rα and OSMRβ cooperate to assemble the receptor complex remains unresolved.

In this study, we report a 3.3 Å cryo-EM structure of the human IL-31/IL-31Rα/OSMRβ complex and delineate how IL-31 engages IL-31Rα at site 2 and OSMRβ at site 3 through distinct receptor-recognition modes. Structure-guided mutagenesis and SPR binding analyses further support the contributions of receptor-side interface residues to IL-31 recognition. Together, these findings establish a structural basis for IL-31 receptor specificity by showing how a non-canonical hydrophilic IL-31–IL-31Rα interface at site 2 and a remodeled IL-31–OSMRβ anchoring network at site 3 distinguish IL-31 receptor engagement from the corresponding receptor-recognition modes used by LIF and OSM.

## Results

### Cryo-EM structure of the human IL-31 receptor complex (hIL-31/hIL-31Rα/hOSMRβ complex)

**Figure 1:**
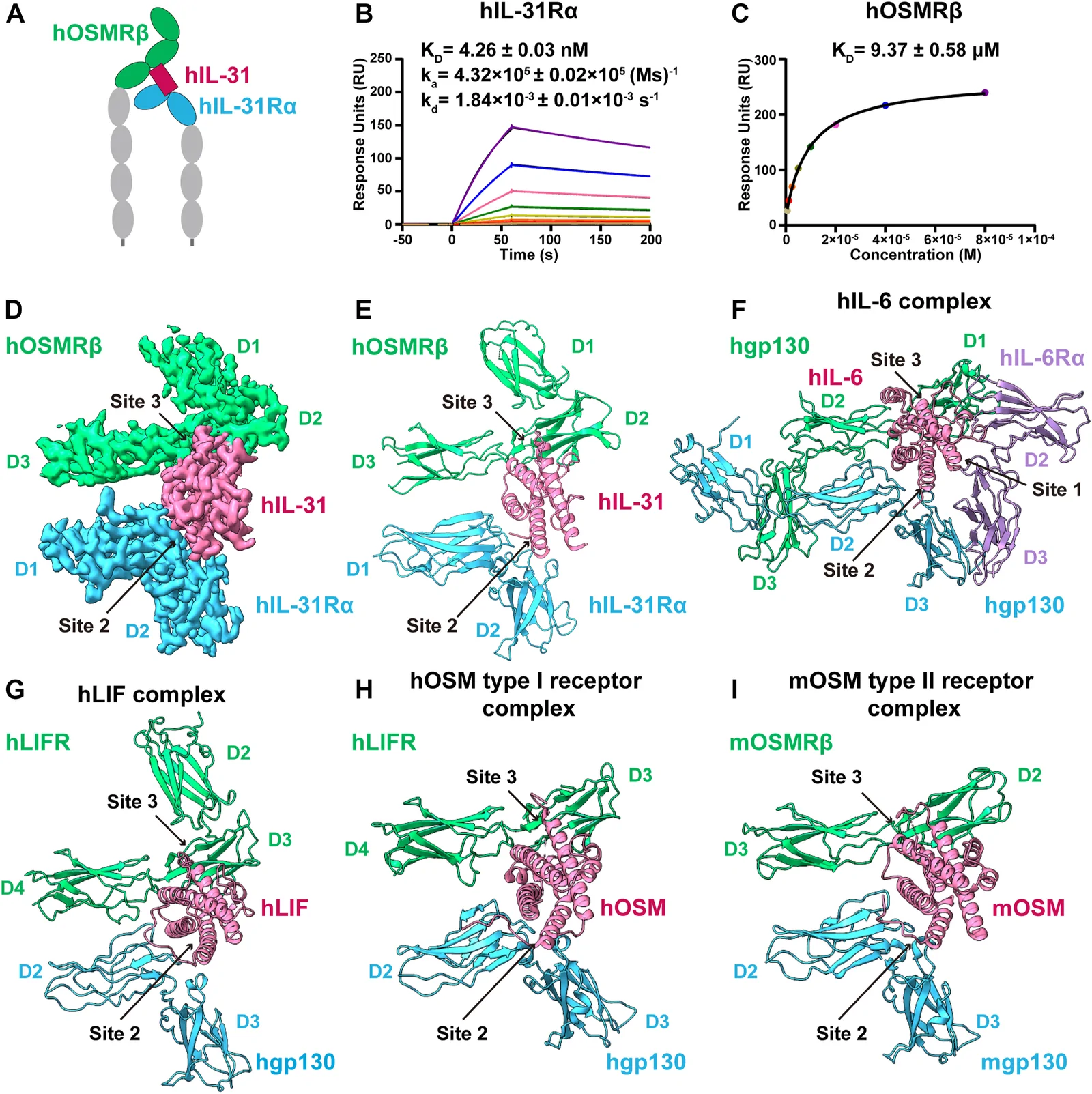
**Structural and biochemical characterization of the human IL-31 receptor complex (hIL-31/hIL-31Rα/hOSMRβ).***A*, cartoon representation of the components of the human IL-31 receptor complex: hIL-31 (*red*), hIL-31Rα (*light blue*), and hOSMRβ (*lime green*), with domains excluded from the imaged constructs shown in *gray*. *B*, SPR analysis of hIL-31Rα binding to immobilized hIL-31. hIL-31Rα was measured using an eight-point, 2-fold serial dilution with a highest analyte concentration of 50 nM. Experimental sensorgrams are shown as colored lines, and global fits to a 1:1 Langmuir binding model are shown as *black lines*. The derived kinetic K_D_, calculated as kd/ka, is indicated together with the fitting SE. *C*, SPR analysis of hOSMRβ binding to immobilized hIL-31. hOSMRβ was measured using an eight-point, 2-fold serial dilution with a highest analyte concentration of 80 μM. Equilibrium binding responses were fitted to a single-site binding model, with the fitted apparent K_D_ ± fitting SE indicated. *D*, refined and sharpened cryo-EM density map of the hIL-31 receptor complex, with hIL-31 colored red, hIL-31Rα colored *light blue*, and hOSMRβ colored *lime green*. *E*, cartoon representation of the atomic model of the hIL-31 receptor complex, colored as in (*D*). *F*, cartoon representation of the hIL-6 receptor complex, containing hIL-6 (*red*), hIL-6Rα (*purple*), and two hgp130 molecules (*light blue* and *lime green*) (PDB: 1P9M). Secondary copies of hIL-6 and hIL-6Rα are omitted for clarity. *G*, cartoon representation of the hLIF receptor complex, containing hLIF (*red*), hgp130 (*light blue*), and hLIFR (*lime green*) (PDB: 8D6A). *H*, cartoon representation of the hOSM type I receptor complex, containing hOSM (*red*), hgp130 (*light blue*), and hLIFR (*lime green*) (PDB: 8V2A). *I*, cartoon representation of the mOSM type II receptor complex, containing mOSM (*red*), mgp130 (*light blue*), and mOSMRβ (*lime green*) (PDB: 8V2C).

To elucidate the structural basis of human IL-31 receptor recognition and assembly (Fig. 1*A*), we purified recombinant hIL-31, hIL-31Rα D1–D2, and hOSMRβ D1–D3 for structural analysis. Size-exclusion chromatography (SEC) analysis showed that the three components formed the hIL-31/hIL-31Rα/hOSMRβ complex upon mixing (Fig. S1). Surface plasmon resonance (SPR) analysis further showed that wild-type hIL-31Rα and hOSMRβ bound immobilized hIL-31 with K_D_ values of 4.26 ± 0.03 nM and 9.37 ± 0.58 μM, respectively (Figs. 1, *B* and *C* and S6*D*). The hIL-31/hIL-31Rα/hOSMRβ complex was characterized by single-particle cryo-EM, yielding 2D class averages representing multiple orientations (Fig. S2*B*). The hIL-31 receptor complex was determined to a resolution of 3.3 Å (Figs. 1*D*, S2, and Table S1). All domains of hIL-31 and hIL-31Rα, as well as the D2–D3 domains of hOSMRβ, exhibited well-resolved cryo-EM density, whereas the D1 domain of hOSMRβ showed weaker density but remained sufficient for reliable model building (Fig. 1*E*).

The hIL-31 receptor complex exhibits a site 2–site 3 architecture similar to the receptor complexes of LIF and OSM, which signal without the requirement for an α-receptor (Fig. 1*E* and *G–I*). Accordingly, these receptor complexes lack a site 1 α-receptor component, in contrast to the IL-6 receptor complex, whose assembly requires engagement of IL-6Rα at site 1 (Fig. 1, *E*–*I*). In this structure, hIL-31 bridges hIL-31Rα and hOSMRβ to form a heterotrimeric receptor assembly. The four-helix bundle of hIL-31 engages the hinge region between hIL-31Rα D1 and D2 to form the site 2 interaction. The posterior face of hIL-31 contacts the D2 and D3 domains of hOSMRβ to form the site 3 interaction (Fig. 1*E*).

### A non-canonical hydrophilic interface mediates hIL-31 recognition by hIL-31Rα

**Figure 2:**
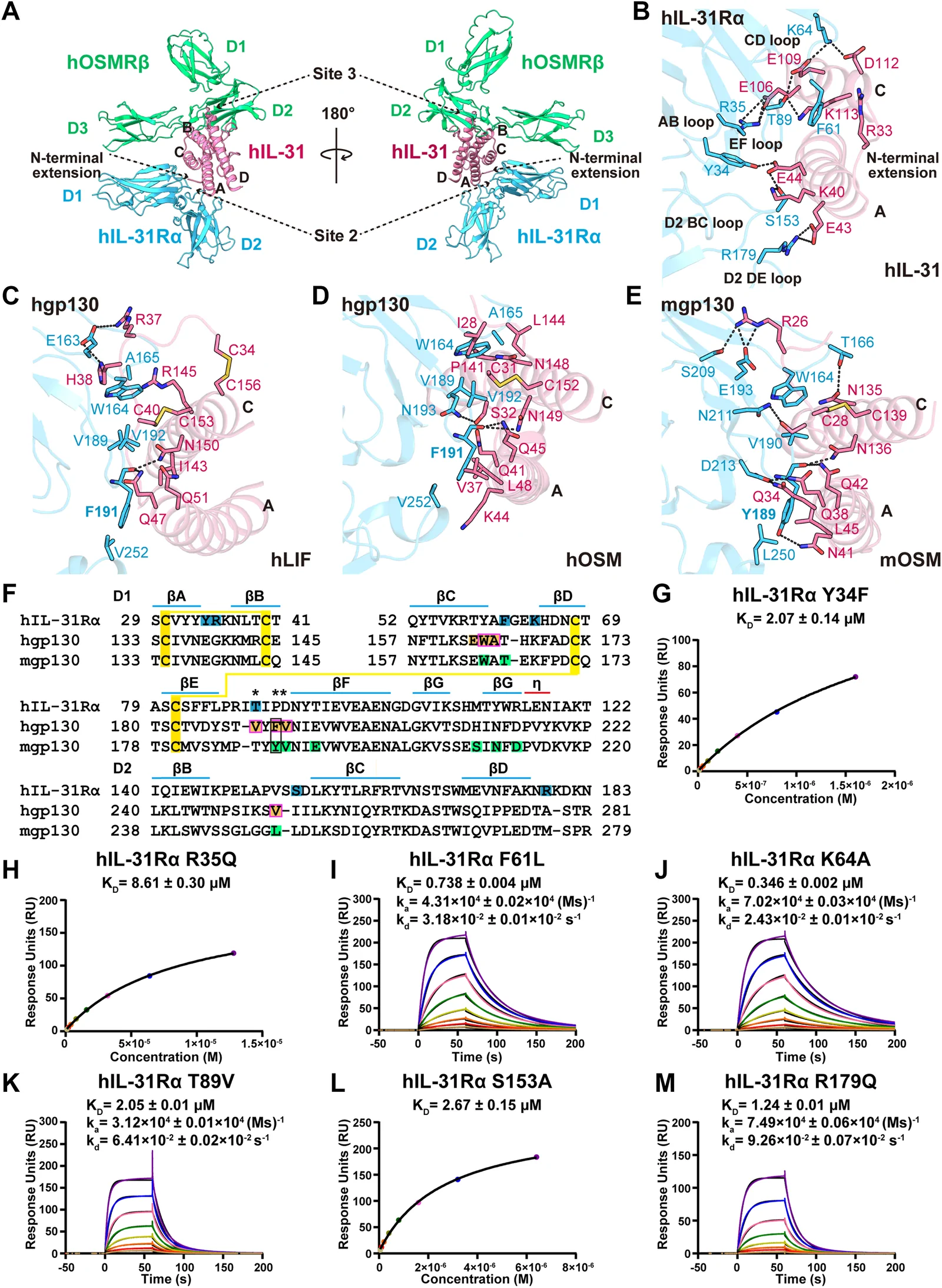
**Site 2 interfaces in IL-31, LIF, and OSM receptor complexes and mutational binding analysis of the IL-31–IL-31Rα interface.***A*, overview of interactions between human IL-31 and its signaling receptors. The complex is shown in cartoon representation and colored by protein components: hIL-31 (*red*), hIL-31Rα (*light blue*), and hOSMRβ (*lime green*). Helix A, helix B, helix C, helix D, the structurally resolved N-terminal extension of hIL-31, and the site 2 and site 3 regions are labeled in the overview. *B–E*, site 2 interfaces of the hIL-31/hIL-31Rα/hOSMRβ complex (*B*), the hLIF/hgp130/hLIFR complex (PDB: 8D6A) (*C*), the hOSM/hgp130/hLIFR complex (PDB: 8V2A) (*D*), and the mOSM/mgp130/mOSMRβ complex (PDB: 8V2C) (*E*). Residues involved in cytokine–receptor interactions are shown as sticks. Disulfide bonds are highlighted in *yellow*. Hydrogen bonds and salt bridges are shown as dashed lines. *F*, sequence alignment of hIL-31Rα, hgp130, and mgp130. Residues involved in the hIL-31Rα–hIL-31 site 2 interface are highlighted in *blue*. hgp130 residues involved in the hLIF site 2 interface are highlighted in *orange*. hgp130 residues involved in the hOSM site 2 interface are boxed in *red*. mgp130 residues involved in the mOSM site 2 interface are highlighted in *green*. The gp130 aromatic anchor residues at site 2 are outlined in *black*. Asterisks mark hIL-31Rα T89, P91, and D92, which were selected for hydrophobic substitutions based on structural superposition and sequence alignment with the corresponding gp130 site 2 regions. *G–M*, SPR analysis of hIL-31Rα mutants binding to immobilized hIL-31. Binding of Y34F (*G*), R35Q (*H*), F61L (*I*), K64A (*J*), T89V (*K*), S153A (*L*), and R179Q (*M*) was measured using eight-point, 2-fold serial dilutions, with the highest analyte concentrations of 1.6, 12.8, 3.2, 1.6, 6.4, 6.4, and 1.6 μM, respectively. Equilibrium binding responses for Y34F, R35Q, and S153A were fitted to a single-site binding model, with the fitted apparent K_D_ ± fitting SE indicated. Experimental sensorgrams for F61L, K64A, T89V, and R179Q are shown as colored lines, and global fits to a 1:1 Langmuir binding model are shown as *black lines*. The derived kinetic K_D_, calculated as kd/ka, is indicated together with the fitting SE.

The site 2 binding interface between hIL-31 and the CHR of hIL-31Rα is primarily mediated by hydrophilic interactions involving helices A and C and the N-terminal extension of hIL-31, together with loops from the D1 and D2 domains of hIL-31Rα (Fig. 2, *A* and *B*). This interaction buries a total surface area of 1607 Å^2^. hIL-31 helix C is primarily engaged by the AB, CD, and EF loops of the hIL-31Rα D1 domain. R35 of the hIL-31Rα D1 AB loop forms multiple salt bridge interactions with E106 of hIL-31 helix C (Figs. 2*B* and S3). An extensive polar interaction network is formed involving K64 of the hIL-31Rα CD loop, T89 of the hIL-31Rα EF loop, and three residues (E109, D112, and K113) within hIL-31 helix C. Specifically, K64 forms salt bridges with E109 and D112, while T89 establishes hydrogen bonds with E109 and K113. The hIL-31 helix A is primarily captured by the D1 AB loop, the D2 BC loop, and the D2 DE loop of hIL-31Rα. A hydrogen-bonding network is formed, in which Y34 of the hIL-31Rα D1 AB loop forms a hydrogen bond with E44 of hIL-31 helix A, while S153 of the hIL-31Rα D2 BC loop forms hydrogen bonds with K40 and E44 of hIL-31 helix A (Fig. 2*B*). R179 of the hIL-31Rα D2 DE loop forms multiple salt bridge interactions with E43 of hIL-31 helix A. In addition, F61 of the hIL-31Rα D1 CD loop forms a cation–π interaction with R33 in the N-terminal extension of hIL-31.

To assess the contribution of interface residues to hIL-31 recognition, we performed structure-guided mutagenesis followed by surface plasmon resonance (SPR) analysis of hIL-31Rα mutants. The hIL-31Rα R35Q mutant was designed to disrupt the R35–E106 electrostatic interaction while preserving side-chain geometry, resulting in an approximately 2000-fold reduction in binding affinity for hIL-31 (Figs. 1*B*, 2*H*, and S6*B*). Although the R35Q substitution was designed to minimize structural perturbation by preserving side-chain geometry, a contribution from mutation-induced local conformational effects cannot be excluded. Consistent with the structural observation, a previous study showed that the ligand-side E106A substitution markedly impaired hIL-31 signaling activity (20), supporting the contribution of the R35–E106 interaction to site 2 recognition. Mutations targeting the helix C-associated polar network (K64A and T89V) reduced binding affinity by approximately 80- and 490-fold, respectively, demonstrating their contributions to hIL-31 recognition (Figs. 1*B* and 2, *J* and *K*). Similarly, Y34F and S153A, which perturb the helix A-associated hydrogen-bonding network, resulted in approximately 490- and 630-fold reductions in affinity, respectively (Figs. 1*B* and 2, *G* and *L*, and S6, *A* and *C*). R179Q, designed to disrupt the E43–R179 salt bridge while preserving side-chain geometry, reduced binding affinity by approximately 290-fold, further supporting the contribution of hIL-31Rα D2-mediated interactions to helix A recognition (Figs. 1*B* and 2*M*). Finally, F61L, which disrupts the F61–R33 cation–π interaction while maintaining local hydrophobic packing, resulted in an approximately 170-fold reduction in affinity, supporting a role for this interaction in stabilizing the hIL-31–hIL-31Rα interface (Figs. 1*B* and 2*I*). Together, these results demonstrate that multiple receptor-side residues within the hIL-31–hIL-31Rα site 2 interface, including R35, T89, Y34, S153, R179, and F61, contribute to high-affinity hIL-31 recognition.

Compared with the LIF and OSM complexes, the site 2 interface of the hIL-31 complex is more hydrophilic (Fig. 2, *B–E*). In the LIF and OSM complexes, gp130 contains a conserved aromatic anchor (hgp130 F191 and mgp130 Y189) that inserts into the hydrophobic face of helix A of LIF or OSM, a feature not observed in the hIL-31 site 2 interface (Fig. 2, *B–F*). To test whether the hIL-31Rα site 2 surface could tolerate a gp130-like local environment, we introduced hydrophobic substitutions at spatially analogous positions in hIL-31Rα, including T89V, P91F, and D92V. Compared with wild-type hIL-31Rα, hIL-31Rα T89V weakened hIL-31 binding by ∼490-fold (Figs. 1*B* and 2*K*). Although P91F and D92V were also designed to introduce gp130-like hydrophobic features, these mutants showed poor biochemical behavior compared with wild-type hIL-31Rα and were therefore not used for quantitative binding interpretation. Together, these findings indicate that the hIL-31Rα site 2 surface does not readily tolerate the tested gp130-like hydrophobic remodeling.

In addition, in LIF and OSM complexes, the N-terminal extension preceding helix A is constrained by disulfide bonds to helix C and contributes additional site 2 contacts for gp130 engagement (Figs. 2, *B–E* and S4) (16, 18). By contrast, the resolved N-terminal region of hIL-31 lacks such a disulfide linkage and contributes only a minor fraction of the ligand-side buried surface area at site 2 (∼7%), compared with ∼42%, ∼29%, and ∼36% for LIF, hOSM, and mOSM, respectively. This limited N-terminal contribution to the site 2 interface is mainly accounted for by the R33–F61 cation–π interaction (Fig. 2*B*). Together, these structural and biochemical analyses show that distributed hydrophilic contacts at site 2, rather than the gp130-type aromatic anchoring mechanism used by LIF and OSM, underlie the receptor-selective engagement of hIL-31Rα by hIL-31.

### A remodeled anchoring mode mediates hIL-31 engagement of hOSMRβ

**Figure 3:**
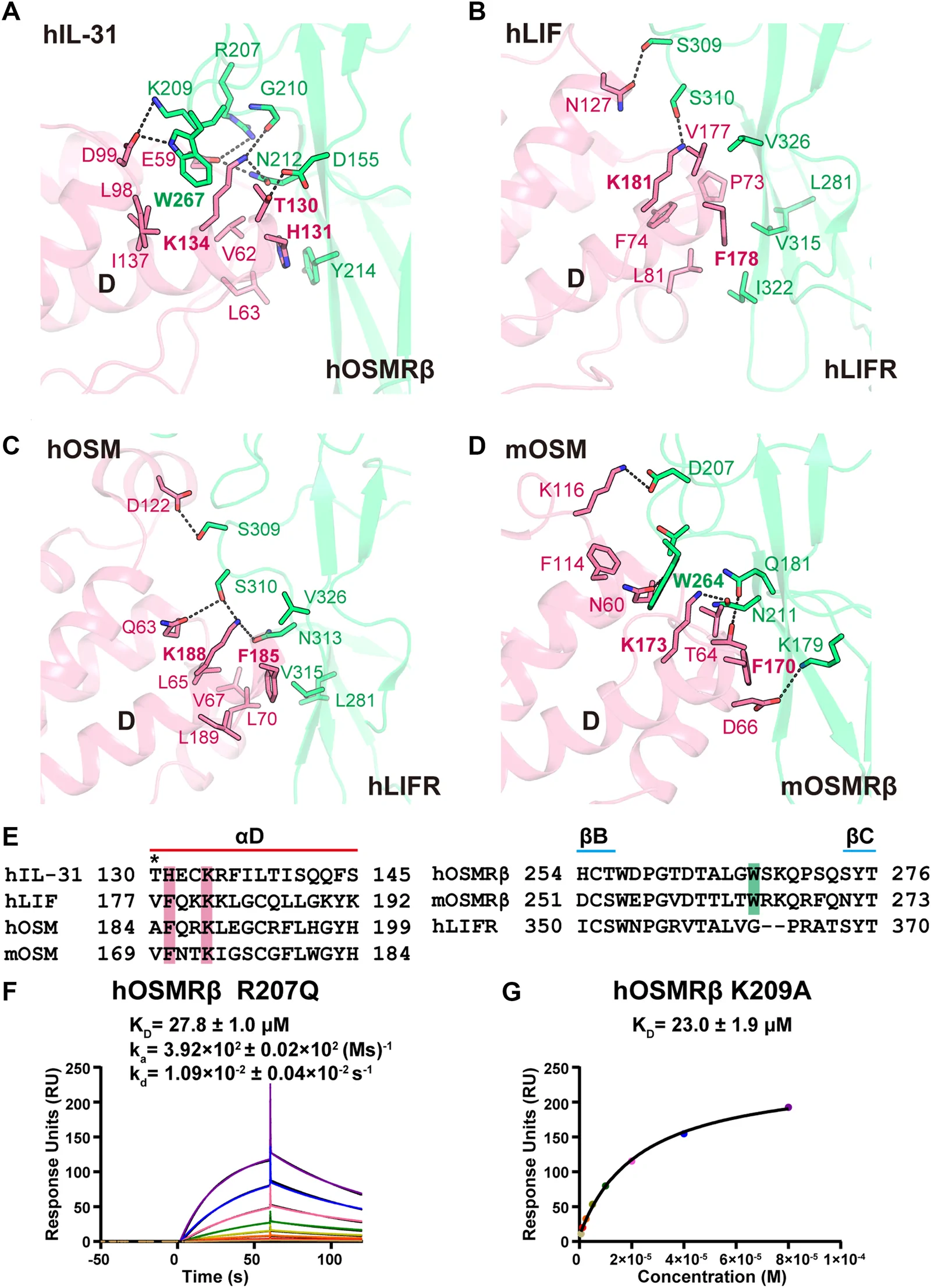
**Site 3 interfaces in IL-31, LIF, and OSM receptor complexes and mutational binding analysis of the IL-31–OSMRβ interface.***A–D*, site 3 interfaces of the hIL-31/hIL-31Rα/hOSMRβ complex (*A*), the hLIF/hgp130/hLIFR complex (PDB: 8D6A) (*B*), the hOSM/hgp130/hLIFR complex (PDB: 8V2A) (*C*), and the mOSM/mgp130/mOSMRβ complex (PDB: 8V2C) (*D*). Residues involved in cytokine–receptor interactions are shown in stick representation. Hydrogen bonds and salt bridges are shown as dashed lines. hIL-31, hLIF, hOSM, and mOSM are colored *red*. hOSMRβ, hLIFR, and mOSMRβ are shown in *lime green*. *E*, sequence alignment of cytokine and receptor interaction regions. Helices and β strands are shown in *red* and *blue lines*, respectively. The FXXK-related motif residues in LIF and OSM, and the corresponding H131 and K134 residues in hIL-31, are highlighted in *red*, whereas the conserved tryptophan residues in hOSMRβ and mOSMRβ are highlighted in *green*. The asterisk indicates the adjacent hIL-31 T130 residue, which forms an additional hydrogen bond with hOSMRβ D155 at the site 3 interface. *F* and *G*, SPR analysis of hOSMRβ mutants binding to immobilized hIL-31. Binding of R207Q (*F*) and K209A (*G*) was measured using eight-point, 2-fold serial dilutions, with the highest analyte concentration of 80 μM. Experimental sensorgrams for R207Q are shown as colored lines, and global fits to a 1:1 Langmuir binding model are shown as *black lines*. The derived kinetic K_D_, calculated as kd/ka, is indicated together with the fitting SE. Equilibrium binding responses for K209A were fitted to a single-site binding model, with the fitted apparent K_D_ ± fitting SE indicated.

hIL-31 is primarily docked onto the D2 Ig domain of hOSMRβ through its helix D and AB loop, with additional contacts between the hIL-31 BC loop and the BC loop of hOSMRβ D3 (Fig. 3*A*), together burying a total surface area of 1312 Å^2^. At the site 3 interface, hIL-31 helix D adopts a binding mode analogous to the FXXK motif region of LIF and OSM, although the conserved phenylalanine is replaced by H131 in hIL-31 (Fig. 3, *A* and *E*). H131 packs against the base of the hOSMRβ D2 Ig domain, whereas the adjacent residue T130 forms an additional hydrogen bond with D155 of hOSMRβ. At the center of the interface, hIL-31 K134 serves as a critical binding anchor through hydrogen bond interactions with N212 and the main-chain carbonyl oxygen of G210 of hOSMRβ (Fig. 3*A*). Consistent with this observation, alanine substitution of hIL-31 K134 has been shown to abolish signaling (20). On one side of the interface, the BC loop of the hOSMRβ D3 domain extends into a cavity formed by helices B and D as well as the AB and BC loops of hIL-31. The hOSMRβ D3 BC loop is longer than the corresponding region in LIFR (Fig. S5), with W267 at its center forming a hydrogen bond with hIL-31 D99 and hydrophobic contacts with hIL-31 L98, I137, and the aliphatic portion of K134. hOSMRβ W267 contributes the largest buried surface area on the receptor side (126 Å^2^), highlighting its central role in the architecture of the site 3 interface. Additional interactions involving the hIL-31 AB and BC loops further stabilize the interface, including E59-mediated hydrogen bond and salt bridge interactions with hOSMRβ N212 and R207, respectively, a hydrophobic interaction between hIL-31 L63 and hOSMRβ Y214, and a salt bridge between hIL-31 D99 and hOSMRβ K209.

To assess the contribution of site 3 residues to hIL-31 recognition, we performed structure-guided mutagenesis followed by SPR analysis of hOSMRβ mutants. D155L, N212V, and W267A were designed to disrupt specific interactions observed in the structure: the D155–T130 hydrogen bond, the N212-mediated hydrogen bonds with hIL-31 K134 and E59, and the W267-mediated hydrogen bond and hydrophobic contacts, respectively. These substitutions were selected to minimize perturbation of the local receptor structure. hOSMRβ D155L, N212V, and W267A showed markedly reduced binding responses that did not permit reliable determination of K_D_ under the tested conditions (Fig. S6, *E*, *G*, and *H*), supporting their important roles in hIL-31–hOSMRβ site 3 recognition. In contrast, hOSMRβ R207Q and K209A retained binding affinities comparable to wild-type hOSMRβ, with approximately 3- and 2.5-fold reductions, respectively (Figs. 1*C* and 3, *F* and *G*, S6*F*). Together, these findings demonstrate that multiple hOSMRβ site 3 residues, particularly W267, N212, and D155, contribute to hIL-31 recognition. The central position of W267 and its extensive receptor-side contacts observed in the structure further support its role as a key component of the anchoring region within the hIL-31–hOSMRβ interface.

The site 3 interface of the hIL-31 complex is topologically similar to those of the LIF and OSM complexes, with the hIL-31 helix D region corresponding to the FXXK motif region of LIF and OSM (Figs. 3, *A–E* and S4). However, the contribution of hIL-31 H131 to site 3 is less pronounced than that of the corresponding phenylalanine residues in hLIF (F178) and hOSM (F185). Alanine substitutions of hLIF F178 and hOSM F185 result in a marked loss of receptor activation, whereas hIL-31 H131A retains detectable signaling activity (20, 21, 22). This difference may be partially explained by the additional contribution of T130 in hIL-31, which forms a hydrogen bond with hOSMRβ D155 at the site 3 interface. At the equivalent positions in LIF and OSM, valine and alanine occupy the corresponding positions, respectively, and lack the hydroxyl group that enables an analogous polar interaction with the receptor (Figs. 3*E* and S4). Notably, OSMRβ participates in site 3 recognition of both IL-31 and OSM, with a conserved tryptophan residue in the BC loop (hOSMRβ W267 and mOSMRβ W264) serving as a conserved structural feature for cytokine–receptor engagement (Figs. 3, *A*, *D* and *E* and S5). Together, these structural and biochemical analyses identify a remodeled site 3 anchoring network in which the T130–D155 hydrogen bond, K134-centered contacts, and the conserved OSMRβ W267 anchor collectively underlie the receptor-selective engagement of hOSMRβ by hIL-31.

## Discussion

The present structure defines the extracellular assembly mechanism of the hIL-31 receptor complex and provides a basis for comparing IL-31 with other IL-6 family cytokine receptor systems. Similar to LIF and OSM, IL-31 assembles its receptor complex through a site 2–site 3 architecture without engagement of an α-receptor at site 1. However, IL-31 uses distinct receptor-recognition features at both interfaces, thereby preserving the broader site 2–site 3 assembly topology shared with LIF and OSM while contributing to receptor-specific recognition.

At site 2, hIL-31 is recognized by hIL-31Rα through an extensive hydrophilic interface rather than the gp130-type aromatic anchoring mode used by LIF and OSM. The limited contribution of the hIL-31 N-terminal region further distinguishes this interface from the disulfide-stabilized N-terminal extension contacts observed in LIF and OSM complexes. Despite its limited contribution to the buried interface, hIL-31 achieves high-affinity recognition through distributed receptor-side interactions. Consistent with this model, receptor-side substitutions within the hIL-31–hIL-31Rα interface impaired hIL-31 binding, and introduction of a gp130-like hydrophobic feature at T89 was poorly tolerated. Together, these findings support the view that IL-31Rα recognizes IL-31 through a receptor-specific hydrophilic surface that is not readily compatible with a gp130-like mode of site 2 engagement.

At site 3, hIL-31 retains the overall anchoring topology of LIF and OSM but remodels the local interaction network. Instead of relying primarily on the canonical phenylalanine within the FXXK motif, hIL-31 uses a composite anchoring arrangement involving K134, the additional T130–D155 interaction, and the conserved hOSMRβ BC-loop tryptophan. This W267-centered feature is consistent with the OSMRβ-mediated anchoring observed in the type II OSM receptor complex (16) and is absent from LIFR-containing complexes, providing a structural basis for the preferential engagement of OSMRβ by IL-31. Thus, IL-31 combines a distinct hydrophilic site 2 interface with a remodeled OSMRβ-mediated site 3 anchoring to contribute to receptor-specific recognition.

The affinity hierarchy observed in our SPR analysis further informs how the IL-31 receptor complex may assemble. IL-31Rα binds hIL-31 with nanomolar affinity through site 2, whereas OSMRβ binds hIL-31 with micromolar affinity through site 3. This hierarchy differs from the hLIF receptor complex, in which site 3 represents the higher-affinity interaction, but is more similar to OSM receptor complexes, in which site 2 provides the higher-affinity interaction (16, 23). In this context, the higher-affinity site 2 interaction is consistent with a site 2-first assembly model, in which IL-31Rα preferentially engages IL-31 before subsequent recruitment of OSMRβ. When projected to the full-length receptor complex, this heterotrimeric extracellular arrangement may influence the relative positioning of the membrane-proximal regions of IL-31Rα and OSMRβ and thereby affect receptor organization. However, because our structure contains only extracellular domains and our biochemical data were obtained using purified proteins, further cell-based studies are required to determine how this extracellular architecture affects receptor activation, signaling strength, and downstream signaling output.

Among the disease-associated variants identified in our literature survey (24, 25, 26), the reported IL-31Rα and OSMRβ variants do not cluster at the cytokine-binding interfaces resolved in our structure, and no reported disease-associated IL-31 variants were found at these interfaces. These observations suggest that currently reported variants are unlikely to directly disrupt the IL-31–IL-31Rα or IL-31–OSMRβ binding contacts visualized here, and additional studies will be needed to define mechanisms involving regions outside the resolved binding interfaces.

Together, our structural and biochemical analyses reveal how a non-canonical hydrophilic site 2 interface and a remodeled OSMRβ-mediated site 3 anchoring network cooperate to confer receptor specificity within the extracellular IL-31 receptor complex while preserving the site 2–site 3 assembly topology shared with LIF and OSM receptor complexes. These findings establish a molecular framework for understanding IL-31 receptor specificity and guiding future structure-based development of IL-31 pathway antagonists.

## Experimental procedures

### Plasmid construction

The coding sequences of human IL-31 (M1–T164; UniProt ID: Q6EBC2), human IL-31Rα (M1–E225; UniProt ID: Q8NI17), and human OSMRβ (M1–Y332; UniProt ID: Q99650) were individually cloned into the pcDNA3.4 expression vector in frame with a C-terminal 6 × His tag. Site-directed mutants were generated from the corresponding wild-type constructs. The IL-31Rα mutants included Y34F, R35Q, F61L, K64A, T89V, P91F, D92V, S153A, R179Q and the OSMRβ mutants included D155L, R207Q, K209A, N212V and W267A. All constructs were verified by Sanger sequencing performed by General Biosystems (Anhui) Corp Ltd before use in subsequent experiments.

### Expression and purification of recombinant human IL-31, IL-31Rα, OSMRβ, and receptor mutants

HEK293F cells were cultured in suspension in 293F Hi-exp medium (OPM Biosciences) at 37 °C with 8% CO_2_ under shaking conditions. Recombinant human IL-31, IL-31Rα, OSMRβ, and their corresponding mutants were transiently expressed using polyethylenimine (PEI; MW 40,000; Cat. no. 24765-1, Kyfora Bio by Polysciences) as the transfection reagent. Each component was expressed and purified separately before *in vitro* reconstitution of the hIL-31/hIL-31Rα/hOSMRβ complex. Cells were transfected at a density of 2 × 10^6^ cells/ml using 1 μg plasmid DNA per milliliter of culture with a DNA:PEI mass ratio of 1:2.5. Four to 5 days after transfection, the culture supernatants were collected and clarified by sequential centrifugation at 760*g* for 15 min to remove cells and at 12,100*g* for 60 min to remove cell debris, followed by filtration. The clarified supernatant was loaded onto a 2 ml Ni-NTA agarose column (QIAGEN). The column was washed with buffer containing 20 mM Tris-HCl, 500 mM NaCl, and 10 mM imidazole, pH 8.0, and bound proteins were eluted with buffer containing 20 mM Tris-HCl, 150 mM NaCl, and 250 mM imidazole, pH 7.4. The eluted proteins were further purified using a Superdex 200 Increase 10/300 Gl column equilibrated in 20 mM Tris-HCl, pH 7.4, and 150 mM NaCl. Fractions containing the target proteins were pooled, flash-frozen, and stored at −80 °C. Protein purity was assessed by SDS-PAGE followed by Coomassie staining.

### Size-exclusion chromatography (SEC)

Size-exclusion chromatography (SEC) was performed using a Superdex 200 Increase 10/300 Gl column (GE Healthcare) equilibrated with 20 mM HEPES and 150 mM NaCl (pH 7.4) at 4 °C. Recombinant hIL-31, hIL-31Rα (D1–D2), and hOSMRβ (D1–D3) were individually injected onto the SEC column to determine their respective elution profiles. To assess complex formation, purified hIL-31, hIL-31Rα (D1–D2), and hOSMRβ (D1–D3) were mixed at a molar ratio of 4:1.2:1, incubated for 1 h at 4 °C, and analyzed by SEC.

### Cryo-EM sample preparation and data collection

An aliquot of 4 μl of the hIL-31/hIL-31Rα/hOSMRβ complex (0.25 mg/ml) was applied to glow-discharged 300-mesh copper grids (Quantifoil R1.2/1.3). The grids were blotted for 3 s with a blotting offset of 0 at 4 °C and 100% humidity and vitrified in liquid ethane using a Vitrobot Mark IV (Thermo Fisher). All cryo-EM datasets were recorded at the Institute of Artificial Intelligence Biomedicine, Nanjing University, on a 300 kV CryoARM300 microscope (JEOL) equipped with an Omega filter (JEOL) and K3 direct electron detector (Gatan), operated in correlated double-sampling mode with SerialEM. Movies were recorded in 50 frames, with a total electron dose of 50 e^−^/Å^2^, an energy filter slit width of 20 eV, and a nominal magnification of 60,000× , corresponding to a calibrated pixel size of 0.803 Å at the specimen level.

### Cryo-EM data processing and model building

Subsequent processing was performed using cryoSPARC v4.7.1 (27). Movies were motion-corrected and contrast transfer functions (CTFs) were estimated. Micrographs with an estimated CTF resolution worse than 5.5 Å or an ice thickness greater than 1.15 were discarded, resulting in 25,111 retained micrographs. These micrographs were subjected to both template-based particle picking and Topaz picking, yielding a total of 5,248,480 particles. Particles were initially extracted with a box size of 288 pixels and binned to 144 pixels. Seven rounds of heterogeneous refinement were performed with one good starting model and five junk models generated by *ab initio* 3D reconstruction during particle screening. Following heterogeneous refinement, 441,650 selected particles were extracted with a box size of 288 pixels without binning and subjected to non-uniform refinement. Subsequently, Rebalance Orientations was applied to improve the particle stack. Global CTF refinement and reference-based motion correction were then performed, yielding a final dataset of 246,851 particles for non-uniform refinement. These particles were further refined using local refinement. The final local refinement yielded a 3D reconstruction at 3.3 Å resolution, estimated using the gold-standard FSC 0.143 criterion with a tight solvent mask in cryoSPARC. The unmasked FSC resolution calculated by the wwPDB validation pipeline based on the deposited half-maps is 3.74 Å, which is more conservative due to the inclusion of solvent noise.

For model building, rigid-body fitting was performed using AlphaFold-predicted models of hIL-31, hIL-31Rα, and hOSMRβ in UCSF ChimeraX (28). The resulting model was refined by real-space refinement in PHENIX (29) and manual building in Coot (30). Structures were analyzed and figures were produced using UCSF ChimeraX (28) or PyMOL (http://www.pymol.org). Buried surface areas of the two binding interfaces were calculated by PDBePISA. Electrostatic surface potentials were calculated using the APBS web server (https://server.poissonboltzmann.org/) and visualized in PyMOL. Sequence alignments were generated using Clustal Omega and manually adjusted based on structural superposition.

### Surface plasmon resonance

For surface plasmon resonance experiments, a Biacore T200 instrument (GE Healthcare Life Sciences) was used. In all experiments, the running buffer consisted of 20 mM HEPES, 150 mM NaCl, and 0.05% Tween 20 (pH 7.4). The CM5 chip surface was activated with N-hydroxysuccinimide (NHS) and N-(3-dimethylaminopropyl)-N′-ethylcarbodiimide hydrochloride (EDC). hIL-31 was diluted to 10 ng/μl in 10 mM sodium acetate (pH 4.5) and immobilized on the activated surface by amine coupling at a flow rate of 10 μl/min for 300 s.

To ensure optimal sample quality for SPR analysis, purified receptor preparations were subjected to size-exclusion chromatography in Biacore running buffer before analysis to remove aggregated species. For wild-type and mutant hIL-31Rα proteins, association was recorded for 60 s, followed by a 180-s dissociation phase, at a flow rate of 30 μl/min and 25 °C. The immobilized hIL-31 surface was regenerated with 12.5 mM NaOH between cycles. The resulting sensorgrams were reference subtracted and analyzed using Biacore T200 Evaluation Software. Sensorgrams for wild-type hIL-31Rα, F61L, K64A, T89V, and R179Q were globally fitted to a 1:1 Langmuir binding model, and kinetic K_D_ values were calculated as kd/ka. For Y34F, R35Q, and S153A, kinetic rate constants could not be reliably determined; therefore, apparent K_D_ and R_max_ values were obtained by steady-state analysis of the equilibrium binding responses using nonlinear fitting to a single-site binding model.

For wild-type and mutant hOSMRβ proteins, association was recorded for 60 s, followed by a 60-s dissociation phase, at a flow rate of 30 μl/min and 25 °C. The immobilized hIL-31 surface was regenerated with 12.5 mM NaOH between cycles. The resulting sensorgrams were reference subtracted and analyzed using Biacore T200 Evaluation Software. Sensorgrams for hOSMRβ R207Q were globally fitted to a 1:1 Langmuir binding model, and the kinetic K_D_ was calculated as kd/ka. For wild-type hOSMRβ and hOSMRβ K209A, the association and dissociation phases were too rapid to permit reliable determination of kinetic rate constants; therefore, apparent K_D_ and R_max_ values were obtained by steady-state analysis of the equilibrium binding responses using nonlinear fitting to a single-site binding model. hOSMRβ D155L, N212V, and W267A showed markedly reduced binding responses that did not permit reliable determination of K_D_ under the tested conditions.

## Data availability

The three-dimensional cryo-EM density map and atomic coordinates of the hIL-31/hIL-31Rα/hOSMRβ complex have been deposited in the EMDB and PDB under accession codes EMD-68584 and 22PL, respectively.

## Supporting information

This article contains supporting information.

## Conflict of interest

The authors declare that they have no conflicts of interest with the contents of this article.

## References

[1] Dillon S.R., Sprecher C., Hammond A., Bilsborough J., Rosenfeld-Franklin M., Presnell S.R. (2004). Interleukin 31, a cytokine produced by activated T cells, induces dermatitis in mice. Nat. Immunol..

[2] Zhang Q., Putheti P., Zhou Q., Liu Q., Gao W. (2008). Structures and biological functions of IL-31 and IL-31 receptors. Cytokine Growth Factor Rev..

[3] Bilsborough J., Leung D.Y., Maurer M., Howell M., Boguniewicz M., Yao L. (2006). IL-31 is associated with cutaneous lymphocyte antigen-positive skin homing T cells in patients with atopic dermatitis. J. Allergy Clin. Immunol..

[4] Feld M., Garcia R., Buddenkotte J., Katayama S., Lewis K., Muirhead G. (2016). The pruritus- and TH2-associated cytokine IL-31 promotes growth of sensory nerves. J. Allergy Clin. Immunol..

[5] Ferretti E., Tripodo C., Pagnan G., Guarnotta C., Marimpietri D., Corrias M.V. (2015). The interleukin (IL)-31/IL-31R axis contributes to tumor growth in human follicular lymphoma. Leukemia.

[6] Kan T., Feldman E., Timaner M., Raviv Z., Shen-Orr S., Aronheim A. (2020). IL-31 induces antitumor immunity in breast carcinoma. J. Immunother. Cancer.

[7] Dreuw A., Radtke S., Pflanz S., Lippok B.E., Heinrich P.C., Hermanns H.M. (2004). Characterization of the signaling capacities of the novel gp130-like cytokine receptor. J. Biol. Chem..

[8] Sonkoly E., Muller A., Lauerma A.I., Pivarcsi A., Soto H., Kemeny L. (2006). IL-31: a new link between T cells and pruritus in atopic skin inflammation. J. Allergy Clin. Immunol..

[9] Cevikbas F., Wang X., Akiyama T., Kempkes C., Savinko T., Antal A. (2014). A sensory neuron-expressed IL-31 receptor mediates T helper cell-dependent itch: involvement of TRPV1 and TRPA1. J. Allergy Clin. Immunol..

[10] Datsi A., Steinhoff M., Ahmad F., Alam M., Buddenkotte J. (2021). Interleukin-31: the "itchy" cytokine in inflammation and therapy. Allergy.

[11] Kabashima K., Matsumura T., Komazaki H., Kawashima M. (2020). Trial of nemolizumab and topical agents for atopic dermatitis with pruritus. New Engl. J. Med..

[12] Kabashima K., Matsumura T., Komazaki H., Kawashima M. (2022). Nemolizumab plus topical agents in patients with atopic dermatitis (AD) and moderate-to-severe pruritus provide improvement in pruritus and signs of AD for up to 68 weeks: results from two phase III, long-term studies. Br. J. Dermatol..

[13] Igarashi A., Katsunuma T., Matsumura T., Komazaki H. (2023). Efficacy and safety of nemolizumab in paediatric patients aged 6-12 years with atopic dermatitis with moderate-to-severe pruritus: results from a phase III, randomized, double-blind, placebo-controlled, multicentre study. Br. J. Dermatol..

[14] Scheller J., Berg A., Moll J.M., Floss D.M., Jungesblut C. (2021). Current status and relevance of single nucleotide polymorphisms in IL-6-/IL-12-type cytokine receptors. Cytokine.

[15] Murakami M., Kamimura D., Hirano T. (2019). Pleiotropy and specificity: insights from the interleukin 6 family of cytokines. Immunity.

[16] Zhou Y., Stevis P.E., Cao J., Ehrlich G., Jones J., Rafique A. (2024). Structures of complete extracellular assemblies of type I and type II oncostatin M receptor complexes. Nat. Commun..

[17] Metcalfe R.D., Hanssen E., Fung K.Y., Aizel K., Kosasih C.C., Zlatic C.O. (2023). Structures of the interleukin 11 signalling complex reveal gp130 dynamics and the inhibitory mechanism of a cytokine variant. Nat. Commun..

[18] Zhou Y., Stevis P.E., Cao J., Saotome K., Wu J., Glatman Zaretsky A. (2023). Structural insights into the assembly of gp130 family cytokine signaling complexes. Sci. Adv..

[19] Boulanger M.J., Chow D.C., Brevnova E.E., Garcia K.C. (2003). Hexameric structure and assembly of the interleukin-6/IL-6 alpha-receptor/gp130 complex. Science.

[20] Le Saux S., Rousseau F., Barbier F., Ravon E., Grimaud L., Danger Y. (2010). Molecular dissection of human interleukin-31-mediated signal transduction through site-directed mutagenesis. J. Biol. Chem..

[21] Deller M.C., Hudson K.R., Ikemizu S., Bravo J., Jones E.Y., Heath J.K. (2000). Crystal structure and functional dissection of the cytostatic cytokine oncostatin M. Structure.

[22] Hudson K.R., Vernallis A.B., Heath J.K. (1996). Characterization of the receptor binding sites of human leukemia inhibitory factor and creation of antagonists. J. Biol. Chem..

[23] Chollangi S., Mather T., Rodgers K.K., Ash J.D. (2012). A unique loop structure in oncostatin M determines binding affinity toward oncostatin M receptor and leukemia inhibitory factor receptor. J. Biol. Chem..

[24] Arita K., South A.P., Hans-Filho G., Sakuma T.H., Lai-Cheong J., Clements S. (2008). Oncostatin M receptor-beta mutations underlie familial primary localized cutaneous amyloidosis. Am. J. Hum. Genet..

[25] Tanaka A., Lai-Cheong J.E., van den Akker P.C., Nagy N., Millington G., Diercks G.F. (2010). The molecular skin pathology of familial primary localized cutaneous amyloidosis. Exp. Dermatol..

[26] Lin M.W., Lee D.D., Liu T.T., Lin Y.F., Chen S.Y., Huang C.C. (2010). Novel IL31RA gene mutation and ancestral OSMR mutant allele in familial primary cutaneous amyloidosis. Eur. J. Hum. Genet..

[27] Punjani A., Rubinstein J.L., Fleet D.J., Brubaker M.A. (2017). cryoSPARC: algorithms for rapid unsupervised cryo-EM structure determination. Nat. Methods.

[28] Pettersen E.F., Goddard T.D., Huang C.C., Meng E.C., Couch G.S., Croll T.I. (2021). UCSF ChimeraX: structure visualization for researchers, educators, and developers. Protein Sci..

[29] Adams P.D., Afonine P.V., Bunkoczi G., Chen V.B., Davis I.W., Echols N. (2010). PHENIX: a comprehensive Python-based system for macromolecular structure solution. Acta Crystallogr. D Biol. Crystallogr..

[30] Emsley P., Cowtan K. (2004). Coot: model-building tools for molecular graphics. Acta Crystallogr. D Biol. Crystallogr..

