# Supplementary material for: Cryo-EM structure of the interleukin-31 receptor complex defines non-canonical interactions that confer receptor specificity
Source: J Biol Chem. 2026 Aug 24;302(10):113472. doi: 10.1016/j.jbc.2026.113472 (PMC13587731; doi:10.1016/j.jbc.2026.113472)
Supplement: Supplementary file 1 [file mmc1.docx]

**Supporting Information**

**Cryo-EM structure of the interleukin-31 receptor complex defines non-canonical interactions that confer receptor specificity**

Yong Feng, Gaofei Qian, Zichu Wei, Xianchi Dong

**Figure S1. Biochemical characterization of the reconstituted hIL-31/hIL-31Rα/hOSMRβ receptor complex.**

(A) Size-exclusion chromatography (SEC) analysis of the reconstituted hIL-31/hIL-31Rα (D1–D2)/hOSMRβ (D1–D3) complex and individual protein components. Peak 1 corresponds to the assembled receptor complex, whereas peaks 2–4 correspond to the individual protein components hOSMRβ (D1–D3), hIL-31Rα (D1–D2), and hIL-31, respectively. (B–E) SDS-PAGE analysis of the indicated SEC fractions and individual protein components under reducing conditions. The corresponding proteins are labeled.

**Figure S2. Cryo-EM data processing workflow of the hIL-31 receptor complex.** (A) Representative cryo-EM micrograph. Scale bar, 50 nm. (B) Representative 2D class averages of the hIL-31 receptor complex. (C) Cryo-EM data processing scheme used to generate the 3D reconstruction of the hIL-31 receptor complex. (D) Orientation distribution of the aligned particles in the final 3D reconstruction output from cryoSPARC. (E) Gold-standard Fourier shell correlation (FSC) curves of the hIL-31 receptor complex reconstruction. FSC curves were calculated without a mask (blue), with a loose mask (green), with a tight mask (red), or with a tight mask followed by high-resolution phase-randomization/noise-substitution correction (purple). The corrected FSC curve supports a global resolution of 3.3 Å according to the gold-standard FSC 0.143 criterion. (F) Local resolution estimation of the final cryo-EM map of the hIL-31 receptor complex. (G) Map–model FSC correlation calculated using PHENIX.

**Figure S3. hIL-31–hIL-31Rα interface.** (A) Cartoon representation of the hIL-31 receptor complex. (B) Open-book representation of the hIL-31–hIL-31Rα interface. Individual proteins are colored as follows: hIL-31 (red), hIL-31Rα (light blue), and hOSMRβ (lime green). (C and D) Coulombic electrostatic surface potential of the hIL-31 receptor complex (C) and the open-book representation of the hIL-31–hIL-31Rα interface (D), calculated based on the structure of the hIL-31 receptor complex. The electrostatic potential is displayed from red (-10 kT/e) to blue (+10 kT/e).

**Figure S4. Structural comparisons of cytokines in the hIL-31, hLIF, hOSM type I, and mOSM type II receptor complexes.** The structures of hIL-31 (A), hLIF (B), hOSM (C), and mOSM (D) are aligned on the four-helix bundle. The FXXK-related site 3 motif regions are shown in stick representations. The additional hIL-31 T130 residue and the corresponding residues at the equivalent positions in hLIF and hOSM (valine and alanine, respectively) are indicated.

**Figure S5. Structural comparison of the BC loops in hOSMRβ (A), hLIFR (B), and mOSMRβ (C).** The corresponding BC-loop regions are boxed in red, and the conserved tryptophan residues in hOSMRβ and mOSMRβ are shown as sticks.

**Figure S6. SPR sensorgrams of hIL-31Rα and hOSMRβ variants binding to immobilized hIL-31.** Binding of hIL-31Rα Y34F (A), R35Q (B), and S153A (C), and hOSMRβ WT (D), D155L (E), K209A (F), N212V (G), and W267A (H) to immobilized hIL-31 was measured using eight-point, 2-fold serial dilutions. The highest analyte concentrations were 1.6 μM for Y34F, 12.8 μM for R35Q, 6.4 μM for S153A, 80 μM for hOSMRβ WT, K209A, and N212V, 20 μM for D155L, and 40 μM for W267A. Representative reference-subtracted sensorgrams are shown. The binding responses of D155L, N212V, and W267A were markedly reduced and did not permit reliable determination of K_D_ under the conditions examined.

**Table S1. Cryo-EM data collection and refinement statistics.**

|  | hIL-31/hIL-31Rα/hOSMRβ complex  (EMD-68584)  (PDB 22PL) |
| --- | --- |
| **Data collection and processing** |  |
| Magnification  Voltage (kV) | 60,000×  300 |
| Electron exposure (e–/Å^2^) | 50 |
| Defocus range (μm) | -0.8 to -2.0 |
| Pixel size (Å) | 0.803 |
| Symmetry imposed | C1 |
| Initial particle images (no.) | 5,248,480 |
| Final particle images (no.) | 246,851 |
| Map resolution (Å) | 3.3 |
| FSC threshold | 0.143 |
| **Refinement** |  |
| Initial model used | AlphaFold |
| Model resolution (Å) | 3.3 |
| FSC threshold | 0.143 |
| Map sharpening B factor (Å^2^) | -108.7 |
| Model composition |  |
| Nonhydrogen atoms | 4,836 |
| Protein residues | 585 |
| Ligand | 7 |
| Mean B factors (Å^2^) |  |
| Protein | 33.5 |
| Ligand | 34.5 |
| R. m. s deviations |  |
| Bond lengths (Å) | 0.003 |
| Bond angles (°) | 0.621 |
| Validation |  |
| MolProbity score | 1.64 |
| Clashscore | 9.31 |
| Poor rotamers (%) | 0.00 |
| Ramachandran plot  Favored (%)  Allowed (%)  Outlier (%) | 97.2  2.8  0.0 |
|  | |

**Table S2. Hydrogen bonds, salt bridges and cation–π interactions at the interfaces in the hIL-31/hIL-31Rα/hOSMRβ complex determined using PyMOL.**

| Site 2 | | | | Site 3 | | | |
| --- | --- | --- | --- | --- | --- | --- | --- |
| hIL-31 | hIL-31Rα | Dist.[Å] |  | hIL-31 | hOSMRβ | Dist.[Å] |  |
| GLU44 [OE1] | TYR34 [OH] | 2.5 | hb | THR130 [OG1] | ASP155 [OD2] | 2.3 | hb |
| GLU109 [OE2] | THR89 [OG1] | 2.8 | hb | LYS134 [NZ] | ASN212 [OD1] | 3.1 | hb |
| LYS113 [NZ] | THR89 [OG1] | 3.2 | hb | LYS134 [NZ] | GLY210 [O] | 3.4 | hb |
| LYS40 [NZ] | SER153 [OG] | 3.4 | hb | ASP99 [OD1] | TRP267 [NE1] | 2.7 | hb |
| GLU44 [OE2] | SER153 [OG] | 2.6 | hb | GLU59 [OE2] | ASN212 [ND2] | 3.0 | hb |
| GLU109 [OE1] | LYS64 [NZ] | 3.1 | sb | GLU59 [OE2] | ARG207 [NH1] | 3.8 | sb |
| ASP112 [OD2] | LYS64 [NZ] | 2.9 | sb | ASP99 [OD1] | LYS209 [NZ] | 3.6 | sb |
| GLU106 [OE2] | ARG35 [NH1] | 2.9 | sb |  |  |  |  |
| GLU106 [OE2] | ARG35 [NH2] | 3.8 | sb |  |  |  |  |
| GLU106 [OE1] | ARG35 [NH1] | 2.5 | sb |  |  |  |  |
| GLU43 [OE1] | ARG179 [NH1] | 3.0 | sb |  |  |  |  |
| GLU43 [OE2] | ARG179 [NH1] | 3.9 | sb |  |  |  |  |
| ARG33 [NE] | PHE61 [centroid] | 4.8 | cp |  |  |  |  |

hb, hydrogen bond; sb, salt bridge; cp, cation–π interaction.

Hydrogen bonds were defined by a donor–acceptor heavy-atom distance (D···A) of ≤3.5 Å. Salt bridges were defined when the distance between oppositely charged atoms was ≤ 4.0 Å. The R33–F61 cation–π interaction was assigned based on proximity between the guanidinium nitrogen and aromatic ring centroid.
